# Indigenous Medicinal Plants—An Important Move-Prize Essays Invited

**Published:** 1881-10

**Authors:** J. C. LeHardy

**Affiliations:** Savannah, Ga.; President of the Medical Association of Georgia


### INDIGENOUS MEDICINAL PLANTS.—AN IMPORTANT MOVE.—PRIZE ESSAYS INVITED.

By J. C. LeHARDY, M.D., Savannah, Ga.
President of the Medical Association of Georgia.

At the last meeting of the State Medical Association, held in Thomasville, the recommendation made by the President, to offer prizes for essays to be competed for by all physicians in the State, was adopted.

Accordingly three prizes are offered. The first of fifty dollars for the best essay on medicinal plants growing in this State, their use in practice, natural history, mode of cultivation and preparation for use and for market. The second and third of twenty-five dollars each, to be awarded for the best essays upon any medical or surgical subject.

In order to carry this project to a successful end, a committee of five has been appointed by the Association. This committee will issue a circular indicating the plan upon which said essays are to be written, thus systematizing and facilitating the work of both writers and committee, and at the same time eliciting the greatest possible amount of practical information upon the subject.

The objects of the Association in offering the first prize are: To disseminate among medical men generally, a knowledge of the virtues of a large number of plants which have been used with great success by some of our country doctors in the treatment of special diseases. This knowledge which is now, so to say, buried and threatens to be lost, will, it is hoped, be brought to light by competing essayists.

To induce country practitioners to keep a record of interesting cases, and make accurate observations upon the effects of the drugs they use.

To create a reciprocity of interest between the country and city doctors, and thus increase the good feeling which should exist in all bodies of men working for the same purpose.

Finally, to establish in the State a new industry—the cultivation and preparation for market of various roots and herbs, which will give a remunerative employment to many of our country people.

Georgia, in common with other Southern States, posseses a remarkably rich flora. Among its numerous medicinal plants, yellow jessamin, pinkroot, blackroot, white and black hellebore, lobelia, etc., have acquired a world-wide reputation, and the value of many others will probably be brought to light by essayists.

The demand for medicinal plants is growing daily with the increase of population, and their cultivation and preparation for market enable many communities, especially in the Middle States, to sustain themselves. As our climate is better adapted to the growth of many of these plants, a better article can be produced here, and of course a better price obtained. Let the people be once convinced of this, and they will readily engage in this industry. But the doctors must give the impulse.

It was originally proposed to make the first prize one hundred dollars, and the second and third fifty dollars each, but as the amount on hand, raised by the subscription of some members of the profession, was not sufficient to make these offers, it was decided to reduce the prizes, while the President was instructed to increase them to the first named amounts, should a sufficient sum be obtained. All members of the profession who desire to assist in this effort—to knit the physicians of the State more closely together and to do something toward developing the resources of Georgia in this direction—are requested to send at once such money as they may feel disposed to subscribe to Dr. Eugene Foster, Augusta, Ga., chairman of the committee on prize essays, in order that the committee may be able to offer such awards to competitors as the sum in hand may warrant.

